# 

**Published:** 1871-04

**Authors:** 


### CONTENTS.

COMMUNICATIONS.

Address to the Graduating Class of Ohio Dental College, March 1, 1871 ..... 143
Mechanical Dentistry ..... 154
Two very Perplexing Cases ..... 158
Advantage of Clinics ..... 160
Things Old and New ..... 161

PROCEEDINGS OF SOCIETIES.

Discussions of the Twenty-seventh Annual Meeting of Mississippi Valley Dental Association ..... 163

SELECTIONS.

The Microscope in Medicine ..... 179
Note on the Physiological Effects of Carbonic Oxide ..... 180
The Production of Asthenia or Anæsthesia in Surgical Operations by Compression of the Vagus Nerves ..... 181
Little Things ..... 182

EDITORIAL.

Mississippi Valley Dental Association ..... 183
"Watt's Elevator"– A Misnomer ..... 184
Educational ..... 185
A Lapse ..... 186
Phosphate of Lime ..... 186
The Treatment of Suppurating Glands of the Neck ..... 187
Obituaries ..... 188

